# Granulomatosis with polyangiitis initially presenting as secretory otitis media: a case report

**DOI:** 10.3389/fsurg.2026.1881731

**Published:** 2026-06-18

**Authors:** Zihan Zhang, Yixi Chen, Yinping Zeng, Zhendong Tang, Jiawei Gu, Wenbo Ma, Tingting Duan, Xuejun Zhou

**Affiliations:** 1 Department of Otolaryngology, Head and Neck Surgery, The First Affiliated Hospital, Hainan Medical University, Haikou, China; 2 Department of Hematology, The First Affiliated Hospital, Hainan Medical University, Haikou, China

**Keywords:** case report, complicated otitis media, facial nerve palsy, granulomatosis with polyangiitis (GPA), secretory otitis media

## Abstract

**Background:**

Granulomatosis with polyangiitis (GPA) is a rare systemic necrotizing vasculitis characterized by granulomatous inflammation of the upper and lower respiratory tracts, glomerulonephritis, and small vessel vasculitis. Otolaryngological manifestations are common in GPA and may precede systemic symptoms, often leading to delayed diagnosis. We report a case of GPA initially presenting as secretory otitis media, which was misdiagnosed and treated as infectious otitis media for a prolonged period.

**Case presentation:**

This study reports a case of atypical GPA in a 50-year-old woman. The patient presented with severe and complicated otitis media, accompanied by hearing loss and facial nerve palsy, as well as ear pain and high fever; however, she did not exhibit any other otolaryngological or systemic clinical manifestations associated with GPA.

**Conclusions:**

Diagnosing GPA is challenging due to its wide range of clinical manifestations. Otological signs and symptoms are relatively rare in this condition, though they may occasionally present as the initial manifestation. Early diagnosis and treatment are crucial for preventing serious and permanent complications caused by this disease. Early recognition and multidisciplinary collaboration are essential to prevent irreversible complications. Otolaryngologists should remain highly vigilant for systemic diseases such as GPA.

## 1 Introduction

Granulomatosis with polyangiitis (GPA), formerly known as Wegener's granulomatosis, is a rare autoimmune systemic necrotizing vasculitis characterized by granulomatous inflammation involving the upper and lower respiratory tracts, kidneys, and small vessels (1). The incidence of GPA is approximately 2–12 cases per million per year, with a slight female predominance in some populations (1). The disease can affect individuals across a broad age range, with peak incidence occurring between the fourth and sixth decades of life (2).

Otolaryngological manifestations represent one of the most common presenting features of GPA, occurring in up to 73% of patients during the course of the disease (3). These manifestations include otitis media, hearing loss, nasal obstruction, epistaxis, sinusitis, subglottic stenosis, and facial paralysis (4). Among otological presentations, conductive hearing loss secondary to otitis media is particularly common and may be the sole initial symptom, frequently leading to misdiagnosis as infectious or inflammatory ear disease (5). The subtle and nonspecific nature of early otological symptoms often results in delayed diagnosis and inappropriate treatment, allowing progression to irreversible tissue damage (6).

Here, we present a case of GPA in a 50-year-old woman whose initial manifestation was secretory otitis media with subsequent facial paralysis, persistent fever, and profound hearing loss. The diagnostic delay and multiple treatment failures underscore the importance of maintaining a high index of suspicion for GPA in patients with refractory otological symptoms, particularly when conventional antimicrobial therapy proves ineffective.

## 2 Case report

A 50-year-old female patient presented to our outpatient clinic with a history of over one month of hearing loss in the left ear accompanied by ear pain. She had no history of major medical conditions such as diabetes, hypertension, or coronary heart disease. She is 158 cm tall and weighs 46 kg. She additionally reported numbness on the left side of her face. There were no other associated nasal, paranasal sinus, or otological symptoms. Of note, she had been diagnosed with left middle ear effusion two months previously at an outside institution, where she underwent myringotomy and tympanostomy tube insertion. She reported minimal improvement in symptoms following this procedure.

On physical examination, both external auditory canals were patent and dry. A left tympanostomy tube was *in situ* without obvious otorrhea; the tympanic membrane surface appeared slightly moist. The right tympanic membrane was intact, dull, and thickened. Neither mastoid region exhibited erythema or tenderness. Nasopharyngeal examination revealed a smooth mucosal surface without mass lesions. Neurological examination was significant for flattening of the left nasolabial fold, incomplete left eye closure, disappearance of left forehead wrinkles, and deviation of the mouth angle to the right, corresponding to House-Brackmann grade IV facial paralysis (7). Rinne tests were positive bilaterally. Weber test lateralized to the right. Schwabach tests were negative bilaterally.

Subsequent otoendoscopy confirmed the left tympanostomy tube in place with slight moisture on the tympanic membrane surface. Transnasal nasopharyngoscopy demonstrated a patent pharyngeal orifice of the Eustachian tube without significant secretions (Figure 1). Tympanometry revealed a type B curve in the right ear. Pure-tone audiometry confirmed bilateral mixed hearing loss, with severe conductive hearing loss in the right ear and profound hearing loss in the left ear (Figure 2).

**Figure 1:**
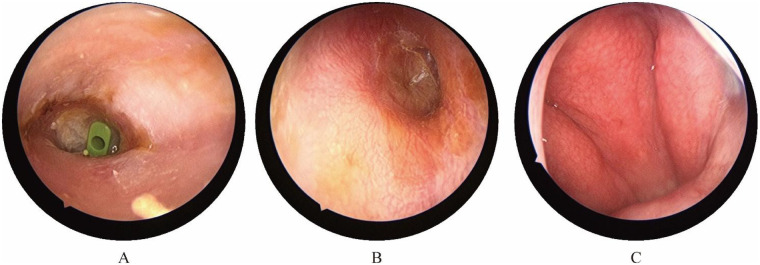
Endoscopic findings of the ear and nasopharynx. **(A)** Otoscopic view of the left ear showing a ventilation tube *in situ* within the tympanic membrane. The surrounding membrane appears slightly retracted and erythematous, with a small amount of cerumen or discharge visible in the external auditory canal. **(B)** Otoscopic view of the right ear reveals an intact but retracted tympanic membrane. The membrane appears opaque and thickened with visible hyperemia, suggestive of middle ear effusion or chronic inflammation, but without evidence of perforation. **(C)** Endoscopic view of the nasopharynx. The image demonstrates the posterior nasal cavity and nasopharyngeal mucosa, which appears smooth and pink, with no obvious signs of mass, hypertrophy of the pharyngeal tonsil, or purulent discharge.

**Figure 2:**
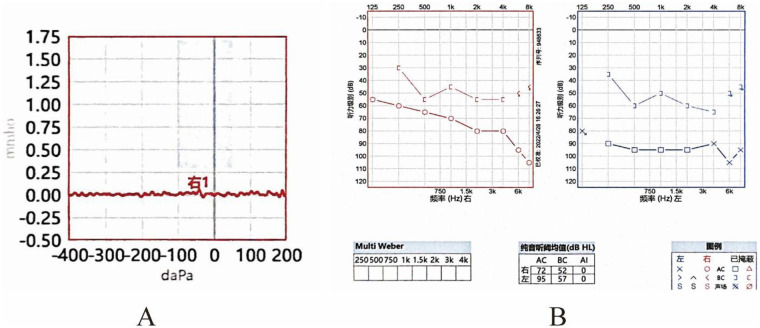
Audiological assessment of the patient: **(A)** tympanometry of the right ear shows a flat (type B) curve, indicating significantly reduced mobility of the tympanic membrane, consistent with middle ear effusion. **(B)** Pure-tone audiometry reveals severe conductive hearing loss in both ears. The right ear demonstrates a mean air-conduction threshold of 72 dB HL and a mean bone-conduction threshold of 52 dB HL. The left ear shows a profound conductive hearing loss, with a mean air-conduction threshold of 95 dB HL and a mean bone-conduction threshold of 57 dB HL. A significant air-bone gap is present bilaterally.

Following admission, laboratory investigations showed a white blood cell count of 9.45 × 10⁹/L with neutrophil predominance (86.5%) and markedly elevated high-sensitivity C-reactive protein (85.84 mg/L). Liver and renal function tests were within normal limits. High-resolution three-dimensional temporal bone computed tomography (CT) demonstrated bilateral mastoiditis with clouding of mastoid air cells, indistinct intercellular septa, and minimal soft tissue density surrounding the intact ossicular chains within the tympanic cavities. Chest CT showed no pulmonary abnormalities. Brain MRI, ear MRI with contrast, and diffusion-weighted imaging revealed white matter hyperintensities in the centrum semiovale and bilateral corona radiata and bilateral mastoiditis (Figures 3, 4).

**Figure 3:**
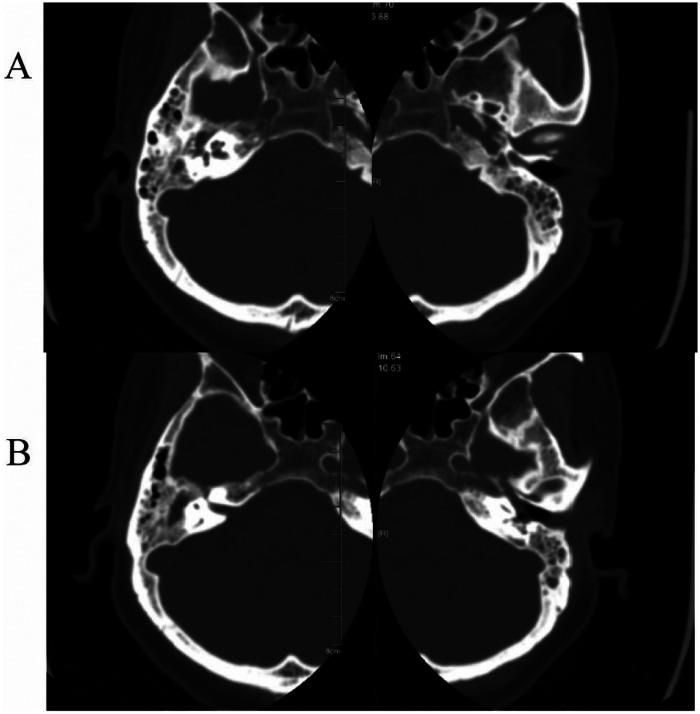
Axial high-resolution computed tomography (HRCT) scans of the temporal bones: **(A)** the right temporal bone scan shows opacification of the middle ear cavity and mastoid air cells. **(B)** The left temporal bone scan reveals more extensive opacification of the middle ear and mastoid air cells, with evidence of bony erosion.

**Figure 4:**
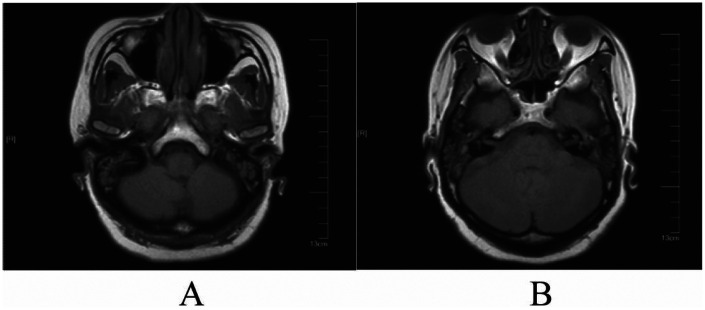
Axial magnetic resonance imaging (MRI) scans of the brain: **(A)** axial T2-weighted image demonstrates bilateral hyperintense signal within the middle ear cavities and mastoid air cells, consistent with fluid accumulation or inflammatory mucosal thickening. **(B)** Axial T1-weighted image shows corresponding hypointense to isointense signal in the same regions, without evidence of abnormal enhancement or intracranial extension.

The patient was initially treated with intravenous methylprednisolone 80 mg and pentoxifylline 0.1 g, supplemented by oral mecobalamin, betahistine, eucalyptol limonene pinene enteric capsules, and levocetirizine. Adjunctive therapies included acupuncture, intranasal budesonide spray, oral valacyclovir, and intravenous clindamycin. Despite this regimen, the patient's otalgia progressively worsened. The left tympanostomy tube was subsequently removed. Two days later, she developed high-grade fever. Following consultation with the department of clinical pharmacy, vancomycin was initiated for presumed refractory infection. However, the fever persisted. Five days thereafter, bilateral myringotomy with tube placement was performed under general anesthesia, resulting in mild alleviation of otalgia. Infectious disease consultation led to a switch to linezolid and oral rifampicin, yet pyrexia continued. Influenza A/B testing and respiratory pathogen IgM panels were negative. Repeat complete blood count showed a white blood cell count of 11.33 × 10⁹/L, neutrophils 76.6%, and persistently elevated high-sensitivity C-reactive protein at 85.54 mg/L.

Rheumatology and immunology consultation was obtained on day 3 of this deteriorating course. Serological testing revealed negative antinuclear antibodies (ANA), anti-neutrophil cytoplasmic antibodies (ANCA) panel, complement levels, and anti-phospholipid antibodies. Erythrocyte sedimentation rate was markedly elevated at 119 mm/h. Six days later, the patient experienced severe otalgia accompanied by headache. The tuberculin skin test (PPD) was negative. Five days subsequently, the fever gradually subsided, but otalgia persisted. Re-examination axial temporal bone HRCT revealed re-aeration of the middle ear cavity and mastoid air cells on the right side, indicating inflammatory resolution. The left ear showed persistent middle ear and mastoid opacification with no obvious improvement compared with the initial scan (Figure 5). Given the absence of IgG4 testing capability at our institution, transfer to another hospital was recommended. Bilateral tympanostomy tubes were removed, and the patient was discharged.

**Figure 5:**
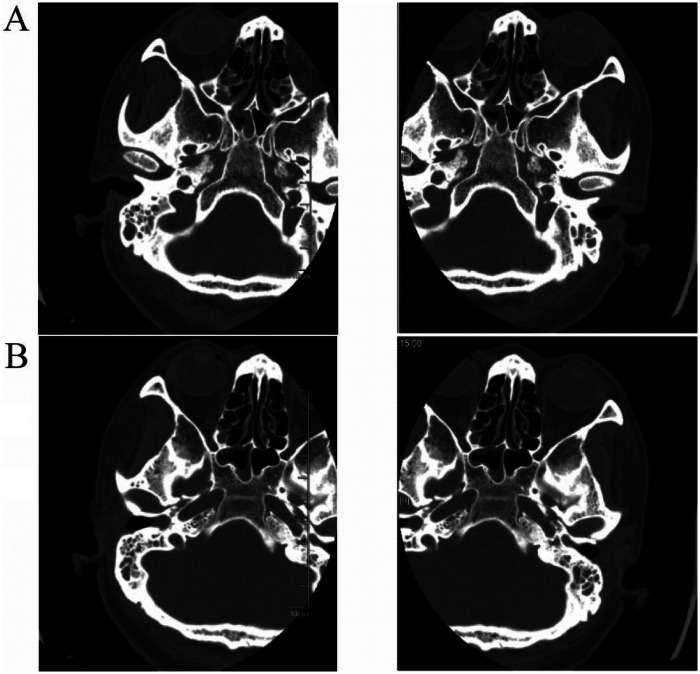
Axial high-resolution computed tomography (HRCT) scans of the temporal bones upon re-examination: **(A)** The right temporal bone demonstrates re-aeration of the middle ear cavity and mastoid air cells, indicating resolution of the inflammation. **(B)** The left temporal bone shows persistent opacification of the middle ear and mastoid air cells, with no significant improvement compared to the initial scan.

At the receiving institution, otoscopy revealed granulation tissue in the left external auditory canal. Transnasal nasopharyngoscopy identified a nasopharyngeal mass. Multiple biopsy specimens were obtained from both the external auditory canal and nasopharynx. Histopathological examination demonstrated fibrogranulomatous inflammation. Based on the characteristic histopathological findings, clinical presentation, and exclusion of infectious etiologies, a diagnosis of granulomatosis with polyangiitis was established. The patient was transferred to the department of rheumatology and immunology, where she received methylprednisolone 500 mg pulse therapy for 3 days, combined with compound sulfamethoxazole and cefoperazone for infection prophylaxis, and rituximab 500 mg.

At follow-up, the patient reported significant relief of bilateral otalgia and marked improvement in trismus. Left-sided facial paralysis persisted. Repeat otoendoscopy and nasopharyngoscopy demonstrated complete resolution of masses in the external auditory canal and nasopharynx. Left-sided hearing showed moderate improvement compared with baseline. Following significant clinical improvement with medical therapy, the patient was discharged with scheduled follow-up appointments in both rheumatology and otorhinolaryngology outpatient clinics.

## 3 Discussion

Granulomatosis with polyangiitis is a systemic small-vessel vasculitis characterized by necrotizing granulomatous inflammation predominantly affecting the respiratory tract and kidneys (1). The disease follows a relapsing-remitting course, and delayed diagnosis is associated with significant morbidity and mortality (8). Otolaryngological involvement occurs in the majority of patients and may constitute the earliest manifestation, frequently preceding systemic disease by months or even years (9).

In the present case, the patient's initial presentation with secretory otitis media and subsequent failure to respond to conventional surgical and antimicrobial interventions represent a classic diagnostic pitfall. Secretory otitis media is one of the most common otological manifestations of GPA, reported in 18%–42% of patients (4, 10). The pathophysiology involves granulomatous inflammation and vasculitis affecting the Eustachian tube and middle ear mucosa, leading to chronic effusion, adhesive changes, and conductive hearing loss (11). Unlike infectious otitis media, GPA-related otitis often proves refractory to antibiotics and myringotomy, as repeatedly observed in our patient (12).

The patient's unilateral facial nerve palsy warrants particular attention; according to the House-Brackmann scale, the patient's facial nerve palsy is classified as Grade IV. Facial nerve involvement in GPA is relatively uncommon, with reported incidence ranging from 1.5% to 5% (13). The mechanism typically involves granulomatous inflammation or vasculitis affecting the facial nerve within the temporal bone, most commonly at the geniculate ganglion or along its mastoid segment (14). The presence of facial paralysis in conjunction with refractory otitis media should raise a strong suspicion for an underlying systemic vasculitis rather than isolated infectious pathology (15).

The diagnostic approach to GPA relies on a combination of clinical presentation, serological markers, histopathological confirmation, and imaging studies (16). Classically, cytoplasmic anti-neutrophil cytoplasmic antibodies (c-ANCA) targeting proteinase 3 are present in 80%–95% of patients with active, generalized GPA (17). However, ANCA negativity does not exclude the diagnosis, as reported in 10%–20% of cases, particularly in limited or early-stage disease (18). Our patient demonstrated negative ANCA, ANA, and complement studies, emphasizing that seronegativity cannot reliably exclude GPA. The markedly elevated erythrocyte sedimentation rate of 119 mm/h, while nonspecific, provided an important clue to ongoing systemic inflammation. Ultimately, histopathological confirmation through biopsy remains the gold standard for diagnosis (19). The finding of fibrogranulomatous inflammation in both the external auditory canal and nasopharynx provided definitive diagnostic evidence.

The absence of renal and pulmonary involvement at presentation is consistent with limited GPA, a variant occurring in approximately 20%–40% of cases (20). Limited GPA is defined by a disease confined to the respiratory tract without overt glomerulonephritis. However, progression to systemic involvement can occur, necessitating vigilant monitoring (21). The negative chest CT and normal renal function in our patient are compatible with this presentation, though long-term follow-up is required to detect potential future organ involvement.

The differential diagnosis for this presentation includes IgG4-related disease, sarcoidosis, tuberculosis, malignant otitis externa, and necrotizing otitis media (22). IgG4-related disease shares histopathological similarities with GPA, including storiform fibrosis and lymphoplasmacytic infiltration, but typically shows elevated serum IgG4 levels and responds differently to corticosteroid therapy (23). The unavailability of IgG4 testing at our institution contributed to the diagnostic delay and necessitated transfer. Tuberculosis was reasonably excluded by negative PPD testing and absence of caseating granulomas on histopathology. Malignant otitis externa, usually caused by Pseudomonas aeruginosa in diabetic patients, was inconsistent with the clinical picture and negative bacterial cultures.

Treatment of GPA involves induction of remission followed by maintenance therapy (24). Standard induction regimens combine high-dose corticosteroids with cyclophosphamide or rituximab (25). Rituximab, a chimeric anti-CD20 monoclonal antibody, has emerged as an effective alternative to cyclophosphamide, particularly in patients with refractory disease or concern for cyclophosphamide toxicity (26). In our patient, methylprednisolone pulse therapy (500 mg daily for 3 days) combined with rituximab 500 mg achieved rapid clinical improvement, resolution of nasopharyngeal and external auditory canal masses, and partial recovery of hearing. The choice of rituximab was appropriate given the patient's refractory course and previous extensive antimicrobial failure. Compound sulfamethoxazole was included for Pneumocystis jirovecii pneumonia prophylaxis, which is recommended during intensive immunosuppression (27).

The persistent left-sided facial paralysis despite effective systemic therapy is a sobering reminder of the irreversible neurological damage that can occur with delayed diagnosis. While some degree of facial nerve recovery is possible with prompt immunosuppressive treatment, established axonal degeneration carries a poor prognosis (28). This underscores the critical importance of early recognition and intervention.

Several lessons emerge from this case. First, GPA should be considered in the differential diagnosis of refractory secretory otitis media, particularly when accompanied by facial paralysis, systemic inflammatory signs, and poor response to antibiotics. Second, ANCA negativity does not exclude GPA; tissue biopsy remains essential for definitive diagnosis. Third, multidisciplinary collaboration between otorhinolaryngology, rheumatology, infectious disease, and pathology is crucial for timely diagnosis and optimal management. Fourth, rituximab-based induction therapy represents an effective strategy for refractory or severe GPA presentations.

## 4 Conclusion

We report a case of granulomatosis with polyangiitis initially presenting as secretory otitis media, complicated by facial paralysis and profound hearing loss. The prolonged diagnostic delay and multiple treatment failures highlight the diagnostic challenges posed by GPA when otological symptoms precede systemic manifestations. Clinicians should maintain a high index of suspicion for GPA in patients with refractory otitis media, particularly in the setting of facial nerve palsy, persistent inflammatory markers, and poor response to conventional antimicrobial therapy. Early histopathological confirmation and multidisciplinary management with immunosuppressive therapy, including rituximab, are essential to prevent irreversible complications and improve patient outcomes.

## Data Availability

The original contributions presented in the study are included in the article/Supplementary Material, further inquiries can be directed to the corresponding authors.
